# Three distinct profiles of barriers to physical activity during pregnancy among Chinese women: a latent profile analysis

**DOI:** 10.3389/fpsyg.2026.1883818

**Published:** 2026-07-23

**Authors:** Ting Shen, Cuiqin Huang, Wei Han, Yan Jiang

**Affiliations:** 1 Shanghai Sixth People’s Hospital Affiliated to Shanghai Jiao Tong University School of Medicine, Shanghai, China; 2 Shanghai Sixth People’s Hospital, Shanghai, China

**Keywords:** anxiety, barriers, latent profile analysis, physical activity, pregnant women, social support

## Abstract

**Introduction:**

Physical activity (PA) is a modifiable factor that contributes to maternal and fetal health. Adherence to the World Health Organization recommendations remains low among pregnant women. Recent studies have identified barriers to PA during pregnancy and the factors influencing it, but population heterogeneity remains underexplored. This study identified distinct profiles of barriers to physical activity during pregnancy and their associated factors, which may facilitate rapid screening of high-risk groups and offer a novel perspective for managing these barriers.

**Methods:**

A descriptive cross-sectional survey was carried out at a general hospital in Shanghai, China, from 1 December 2024 to 31 May 2025. Three hundred pregnant women completed a demographic characteristics questionnaire, the social support rating scale, the Generalized Anxiety Disorder-7 (GAD-7) questionnaire, and the barriers to physical activity during pregnancy scale (BPAPS). Latent profile analysis (LPA) was used to identify groups showing different levels of barriers to physical activity during pregnancy. Additionally, univariate analysis and multinomial logistic regression analysis were used to reveal the factors influencing the different groups.

**Results:**

Three profiles of barriers to PA during pregnancy emerged: the “Low-barrier group” (10.3%), the “Moderate-barrier group” (54.3%), and the “High-barrier group” (35.3%). Multinomial logistic regression analysis showed that income, pre-pregnancy exercise frequency, exercise guidance, and the GAD-7 score were significantly associated with profile membership.

**Discussion:**

This study categorized barriers to PA during pregnancy and identified their associated factors. Healthcare providers may use these findings to deliver tailored interventions to reduce these PA barriers.

## 1 Introduction

Physical activity (PA) refers to any bodily movement produced by skeletal muscle contraction that results in energy expenditure and is essential for health maintenance (Caspersen et al., 1985). Regular PA during pregnancy benefits both the mother and the fetus (Bull et al., 2020). It can significantly reduce the risk of gestational hypertension, gestational diabetes mellitus, excessive weight gain, perinatal depression, low back pain, emergency cesarean section, and neonatal complications (Laredo-Aguilera et al., 2020; Meander et al., 2021; Melzer et al., 2010), and also improves muscle strength, labor pain, postpartum weight recovery, and fetal oxygen supply (Downs et al., 2012). PA is a modifiable factor for maternal and fetal health (Meander et al., 2021).

The recognition of PA as a vital sign has reshaped prenatal care (Sallis et al., 2016). The World Health Organization (WHO) recommends at least 150 min of moderate-intensity PA per week for pregnant women without contraindications or complications (Bull et al., 2020). A survey conducted in Shanghai showed that 47.5% of pregnant women experienced physical inactivity, and only 2.8% met the recommended duration (Zhou et al., 2022). This low adherence may be partly because walking, a light-intensity activity, was the predominant form of PA, and some pregnant women and healthcare workers remain anxious about the risks of moderate-to-vigorous PA. Another study found that the prevalence of physical inactivity was highest (66.2%) in the first trimester (Xiang et al., 2024). The percentages of pregnant women in Jordan, Alabama and Rhode Island (USA), and Zimbabwe who met the WHO recommendations were 28.9, 8.8, and 11%, respectively (Abutalib et al., 2026; Alhemedi et al., 2025; Hove et al., 2025).

Barriers to PA are defined as obstacles that hinder individuals from initiating, maintaining, or increasing PA (Zaragoza et al., 2011). Barriers to PA include intrapersonal barriers (e.g., fatigue, nausea, lack of motivation, and pain), interpersonal barriers (e.g., advice from others and unclear guidance), and environmental factors (e.g., weather conditions; Castilla et al., 2026; He et al., 2023; May et al., 2024; Shang et al., 2023). BPAPS, developed by Amiri-Farahani et al. (2021), assesses these domains. Mean BPAPS scores were 88.55 in Iran and 85.35 in Ibadan (Dolatabadi et al., 2022; Suberu and Adeoye, 2024). Pre-pregnancy body mass index, antenatal admission, education, parity, exercise attitudes, ethnicity, income, and pre-pregnancy exercise habits were associated with PA barriers (Ahmadi et al., 2021; Dolatabadi et al., 2022; Shang et al., 2023; Suberu and Adeoye, 2024).

In summary, existing studies have described PA barriers and their correlates while neglecting population heterogeneity, which remains underexplored. Latent profile analysis (LPA) is a person-centered approach that identifies distinct subgroups based on participants’ responses to individual items, allowing for the exploration of population heterogeneity (Tao et al., 2025). To our knowledge, no previous study has used LPA to identify distinct subgroups of pregnant women based on their barriers to PA. Consequently, this study aimed to (1) classify pregnant women into subgroups based on distinct profiles of barriers to PA and (2) explore the correlates of barriers to PA across different potential profiles.

## 2 Methods

### 2.1 Study population

This descriptive cross-sectional study followed the Reporting of Observational Studies (STROBE) checklist (Vandenbroucke et al., 2007). The participants were recruited from a tertiary hospital in Shanghai, China. The inclusion criteria were as follows: (1) provided informed consent and voluntarily to participate, (2) had adequate cognitive and behavioral abilities, and (3) were aged ≥18 years. The exclusion criteria were as follows: (1) had absolute contraindications to PA (e.g., preeclampsia, cervical insufficiency, or unexplained persistent vaginal bleeding). To ensure reliable and precise subgroup results in LPA, a minimum of 300 participants was necessary according to the recommendation (Ferguson et al., 2020). Hair et al. (2010) suggested that the sample size in LPA should be 5–10 times the number of variables. Allowing for 10% invalid responses, the total required sample size ranged from 160 to 319 on a 29-item scale. A total of 320 questionnaires were distributed to pregnant women from 1 December 2024 to 31 May 2025. Convenience sampling was adopted in this study, which may constitute a potential source of selection bias. Ultimately, we received 300 valid responses, resulting in an effective response rate of 93.8%.

### 2.2 Measures

#### 2.2.1 General information questionnaire

The demographic characteristic questionnaire included age, gestational age, current employment status, education level, personal monthly income, frequency of pre-pregnancy exercise, duration of each pre-pregnancy exercise session, and exercise guidance. These variables were selected as potential confounders based on previous literature and clinical experience.

#### 2.2.2 Barriers to physical activity during pregnancy scale (BPAPS)

Barriers to physical activity during pregnancy were measured using the 29-item self-reporting BPAPS by Amiri-Farahani et al. (2021). BPAPS includes four dimensions: intrapersonal barriers related to pregnancy, intrapersonal barriers unrelated to pregnancy, interpersonal barriers, and environmental barriers. The BPAPS was scored on a 5-point Likert scale: 5 = strong agreement, 4 = agreement, 3 = neutral, 2 = disagree, and 1 = strong disagreement. The scale demonstrated good internal consistency; the Cronbach’s *α* coefficient of the overall scale was 0.824, and the Cronbach’s α coefficients of the dimensions ranged from 0.722 to 0.815. The Chinese version of BPAPS was developed by Yang et al. (2024). The Cronbach’s *α* coefficient for this study was 0.946.

#### 2.2.3 Social support rating scale

The level of social support was measured using a ten-item self-report social support rating scale developed by Xiao (1994). The scale includes three subscales: objective support (three items), subjective support (four items), and support utilization (three items). Items 1–4 and 8–10 are rated on a 4-point Likert scale. Question 5 has five options (A–E), and the total score is calculated based on each option from “none” (1 point) to “full support” (4 points). For questions 6 and 7, the answer “from the following sources” scores a few points if there are several sources, while “no source” responses receive 0 points. Total scores range from 12 to 66, categorized as low (≤22), moderate (23–44), or high (≥45). The Cronbach’s *α* coefficient for this study was 0.769.

#### 2.2.4 Generalized Anxiety Disorder-7 (GAD-7) questionnaires

Anxiety was measured using the GAD-7 questionnaire, which was developed to measure the level of anxiety, has been validated in primary care settings (Spitzer et al., 2006), and is widely used in various populations (Plummer et al., 2016). The scale consists of seven items, each with four options, and the scores range from 0 (not at all) to 3 (almost every day), with higher scores indicating more severe anxiety symptoms in the study participants. The Cronbach’s *α* coefficient for this study was 0.925.

### 2.3 Data collection

Data were collected online via the “Questionnaire Star” platform,^1^ a popular online data-gathering tool in China used for conducting web-based surveys. One researcher met with the participants face-to-face to explain the purpose of the questionnaire. After obtaining informed consent, the researcher provided a quick response (QR) code for participants to scan and complete the questionnaire. After completion, two researchers checked each questionnaire for completeness and data integrity. The platform allowed only one response per account to prevent duplicate submissions.

### 2.4 Ethical considerations

Questionnaires were completed anonymously to ensure confidentiality and to reduce participants’ concerns, thereby enhancing the authenticity and reliability of the responses. An online version of the informed consent form was provided to all participants before they completed the questionnaire. Participants were assured of their right to withdraw at any time, and all data were used solely for the purpose of this study and kept strictly confidential. This study was approved by the Ethics Committee [2025-KY-148 (K)].

### 2.5 Data analysis

We performed LPA using Mplus 8.0 based on the 29 BPAPS items. Model fit was evaluated using the Akaike information criterion (AIC), Bayesian information criterion (BIC), adjusted BIC (aBIC), entropy, bootstrap likelihood ratio test (BLRT), and Lo–Mendell–Rubin likelihood ratio test (LMRT). Lower AIC, BIC, and aBIC values indicated better fit (Peugh and Fan, 2013; Tofighi and Enders, 2007). Entropy values closer to 1 indicated better classification accuracy. Significant LMRT and BLRT results (*p* < 0.05) indicated that the K-class model was superior to the K-1 class model (Choi et al., 2019; Nylund et al., 2007). The final model was selected based on both statistical indices and interpretability. Descriptive analyses were conducted using SPSS 26.0. Continuous variables were presented as mean ± standard deviation, and categorical variables as frequencies and percentages. No adjustment for multiple comparisons was applied to the descriptive comparisons in Table 1, as these analyses were exploratory (Rothman, 1990).

**Table 1:** Demographic characteristics of participants across the three latent profiles (*n*, %).

Characteristic	Total number (*n* = 300)	Profile 1 (*n* = 31)	Profile2 (*n* = 163)	Profile 3 (*n* = 106)	*χ*^2^/*F*	*p*-values
Age, years					0.094	0.955
≤35	246 (82)	26 (83.9)	133 (81.6)	87 (82.1)		
>35	54 (18)	5 (16.1)	30 (18.4)	19 (17.9)		
Gestational age					3.334	0.189
<28 weeks	35 (11.7)	2 (6.5)	24 (14.7)	9 (8.5)		
≥28 weeks	265 (88.3)	29(93.5)	139 (85.3)	97 (91.5)		
Employment status					0.253	0.881
Unemployed	47 (15.7)	5 (16.1)	24 (14.7)	18 (17.0)		
Employed	253 (84.3)	26 (83.9)	139 (85.3)	88 (83.0)		
Education level					7.339	**0.025**
High school or below	210 (70)	17 (54.8)	110 (67.5)	83 (78.3)		
College or above	90 (30)	14 (45.2)	53 (32.5)	23 (21.7)		
Monthly personal income (CNY)					7.412	**0.025**
≤10,000	108 (36)	10 (32.3)	49 (30.1)	49 (46.2)		
>10,000	192 (64)	21 (67.7)	114 (69.9)	57 (53.8)		
Pre-pregnancy exercise frequency					26.463	**<0.001**
None	70 (23.3)	1 (3.2)	29 (17.8)	40 (37.7)		
1~2 times/week	113 (37.7)	11 (35.5)	67 (41.1)	35 (33.0)		
3~6 times/week	61 (20.3)	10 (32.3)	34 (20.9)	17 (16.0)		
Daily	56 (18.7)	9 (29.0)	33 (20.2)	14 (13.2)		
Duration per pre-pregnancy exercise session					8.454	0.076
≤30 min	191 (63.7)	15 (48.4)	99 (60.7)	77 (72.6)		
30–60 min	88 (29.3)	14 (45.2)	50 (30.7)	24 (22.6)		
>60 min	21 (7)	2 (6.5)	14 (8.6)	5 (4.7)		
Received exercise guidance during pregnancy					6.233	**0.044**
No	260 (86.7)	28 (90.3)	134 (82.2)	98 (92.5)		
Yes	40 (13.3)	3 (9.7)	29 (17.8)	8 (7.5)		
Social support rating scale score	41.01 ± 6.87	44.23 ± 6.46	41.66 ± 6.67	39.08 ± 6.80	8.779	**<0.001**
GAD-7 score	4.42 ± 2.07	3.61 ± 1.34	3.85 ± 1.45	5.54 ± 1.65	6.403	**0.002**

Data are presented as *n* (%) for categorical variables and mean ± standard deviation (SD) for continuous variables.

*χ*^2^ = chi-squared test (for categorical variables); *F* = one-way ANOVA (for continuous variables). Bold *p*-values indicate statistical significance (*p* < 0.05).

Profile definitions: Profile 1-Low-barrier group; Profile 2-Moderate-barrier group; Profile 3-High-barrier group.

GAD-7, Generalized Anxiety Disorder 7-item (Scale).

Multinomial logistic regression was performed using Stata 15.0 (mlogit) to identify factors associated with latent profile membership (baseline: Profile 2), with robust standard errors to account for heteroskedasticity. Variables that were significant in univariate analyses (education level, monthly personal income, pre-pregnancy exercise frequency, received exercise guidance, social support rating scale score, and GAD-7 score) were entered as independent variables in the multinomial logistic regression model. The results are reported as odds ratios (OR) with 95% confidence intervals (CI). Statistical significance was set at *p* < 0.05 (two-sided).

Given that multinomial logistic regression assumes independence of irrelevant alternatives (IIA), we attempted the Hausman–McFadden test. However, due to the small sample size in Profile 1 (*n* = 31, 10.3%), the test had insufficient statistical power (Long and Freese, 2005). Therefore, we conducted multinomial probit regression as a sensitivity analysis, which does not require the IIA assumption. The two models yielded consistent results for key predictors, confirming the robustness of our findings (Appendix Table A1).

## 3 Results

### 3.1 Participants characteristics

After quality and integrity checks, 300 valid questionnaires were analyzed. Among the included participants, 18% were aged >35 years. The majority of the participants had a gestational age of ≥28 weeks (88.3%) and were employed (84.3%). Seventy percent had a high school education or below; 64% had a monthly income >10,000 yuan; 23.3% had never exercised before pregnancy, 63.7% exercised less than 30 min per session before pregnancy, and 86.7% had never received PA guidance. The mean scores for the social support rating scale and GAD-7 were 41.01 and 4.42, respectively. Detailed information is provided in Table 1.

### 3.2 LPA results of barriers to physical activity during pregnancy scale

The mean item score for the total BPAPS was 2.51 ± 0.60. The mean item scores for the four subscales (intrapersonal barriers related to pregnancy, intrapersonal barriers unrelated to pregnancy, interpersonal barriers, and environmental barriers) were 2.87 ± 0.73, 2.47 ± 0.78, 2.14 ± 0.70, and 2.34 ± 0.67, respectively. We fitted models with one to four profiles; results are shown in Table 2. The results show that as the number of categories gradually increased, the AIC, BIC, and aBIC for models 1–4 decreased. However, in the four-category model, the LMRT value (0.6141) was not significant. In the three-category model, the entropy value (0.964) was higher, and the LMRT and BLRT values were significant. In addition, the diagonal values of the average latent class probability matrix were all >0.70, indicating good classification reliability (Table 3). Therefore, three profiles were selected as best-fit models.

**Table 2:** Fitting index of latent profile analysis about the participants’ barriers to physical activity during pregnancy scale.

Model	AIC	BIC	aBIC	Entropy	LMRT	BLRT	Class probability
1	23637.361	23852.180	23668.238	—	—	—	—
2	21584.593	21910.526	21631.442	0.941	0.0007	<0.001	155/145 (0.52/0.48)
3	20707.097	21144.143	21144.143	0.964	0.0163	<0.001	31/163/106 (0.10/0.54/0.35)
4	20267.819	20815.979	20346.610	0.957	0.6141	<0.001	33/119/66/82 (0.11/0.39/0.22/0.28)

AIC, Akaike information criterion; BIC, Bayesian information criterion; aBIC, sample size-adjusted Bayesian information criterion; BLRT, bootstrap likelihood ratio test; LMR, Lo–Mendell–Rubin adjusted likelihood ratio test.

**Table 3:** Average attribution probability matrix for each potential profile.

Potential profile	1	2	3
1	**0.976**	0.024	0.000
2	0.000	**0.989**	0.011
3	0.000	0.024	**0.976**

Diagonal values (in bold) of the average latent class probability matrix were all > 0.70, indicating good classification reliability.

### 3.3 Latent category and characteristics of barriers to physical activity during pregnancy among the participants

**Figure 1:**
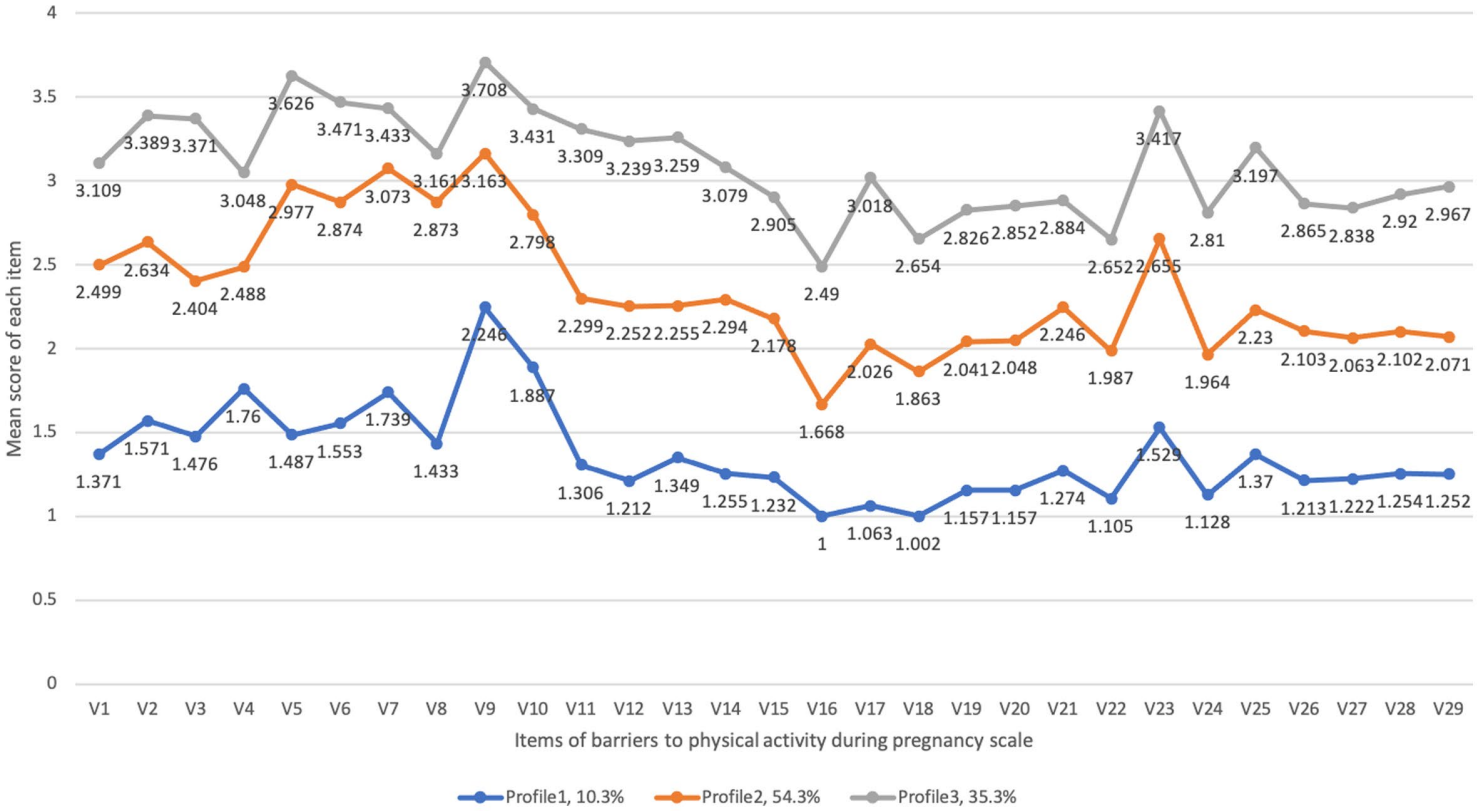
Latent profile of the barriers of PA among pregnant women.

Based on the latent classification results, Figure 1 presents the item-response patterns for the three profiles. The total score for Profile 1 was 39.23 ± 7.329, and the average score for all items was approximately 1.35 points, indicating a low level of barriers to physical activity during pregnancy. Therefore, this category was named the “Low-barrier group.” This group comprised 31 participants (10.3%). The total score for Profile 2 was 68.11 ± 7.053, and the average score for all items was approximately 2.35 points, showing a moderate level of barriers to physical activity during pregnancy. We labeled Profile 2 as the “Moderate-barrier group.” There were 163 participants in Profile 2 (54.3%). The total score for Profile 3 was 90.02 ± 9.876, and the average score for all items was approximately 3.10 points, indicating a high level of barriers to physical activity during pregnancy. Therefore, Profile 3 was named the “High-barrier group” and comprised 106 participants in Profile 3 (35.3%).

### 3.4 Univariate analysis and multinomial logistic regression analysis of factors influencing latent profile membership

Univariate analyses revealed that education level, monthly personal income, pre-pregnancy exercise frequency, received exercise guidance, social support rating scale score, and GAD-7 score were significantly associated with the three latent profiles (*p* < 0.05; Table 1). These variables were then entered as independent variables into a multinomial logistic regression model, with Profile 2 (“Moderate-barrier group”) as the reference category, to identify independent predictors of profile membership. The model demonstrated good overall fit (LR *χ*^2^ = 56.45, df = 16, *p* < 0.001; Pseudo *R*^2^ = 0.1096).

Compared with the moderate-barrier group, none of the independent variables emerged as statistically significant predictors of membership in the Low-barrier group (all *p* > 0.05). The detailed results are presented in Table 4.

**Table 4:** Multinomial logistic regression: factors associated with latent profile membership (baseline: Profile 2, moderate-barrier group).

Predictors	Profile 1 vs. Profile 2			Profile 3 vs. Profile 2		
	*β*	OR [95% CI]	*p*	*β*	OR [95% CI]	*p*
Education (ref: high school or below)						
College or above	0.505	1.657 [0.759, 3.616]	0.205	−0.375	0.687 [0.366, 1.289]	0.242
Income (ref: ≤10,000 yuan)						
>10,000 yuan	−0.360	0.698 [0.284, 1.713]	0.432	−0.625	0.535 [0.305, 0.939]	**0.029**
Pre-pregnancy exercise frequency (ref: no exercise)						
1–2 times/week	1.642	5.164 [0.653, 40.838]	0.120	−0.826	0.437 [0.228, 0.840]	**0.013**
3–6 times/week	2.068	7.912 [0.974, 64.285]	0.053	−0.716	0.489 [0.221, 1.080]	0.077
Daily	1.936	6.928 [0.790, 60.785]	0.081	−1.102	0.332 [0.141, 0.782]	**0.012**
Received exercise guidance (ref: no guidance)						
Yes	−0.802	0.448 [0.120, 1.682]	0.234	−1.011	0.364 [0.159, 0.831]	**0.016**
Social support rating scale score	0.051	1.052 [0.989, 1.119]	0.105	−0.042	0.959 [0.919, 1.001]	0.058
GAD-7 score	−0.006	0.994 [0.872, 1.134]	0.933	0.084	1.088 [1.019, 1.161]	**0.011**
Constant	−5.350	0.005 [0.000, 0.077]	<0.001	2.096	8.138 [1.252, 52.872]	0.028
Observations		300				
Log-likelihood		−249.3651				
Pseudo *R*^2^		0.1096				
LR *χ*^2^ (df)		56.45(16)				
*p*		<0.001				

The multinomial logistic regression model demonstrated a good overall fit [LR *χ*^2^(16) = 56.45, *p* < 0.001]. Bold values indicate statistical significance at *p* < 0.05.

Reference group: Profile 2 (moderate-barrier group).

OR, odds ratio; 95%CI, 95% confidence interval.

GAD-7, Generalized Anxiety Disorder 7-item (Scale).

Several factors were significantly associated with distinguishing Profile 3 from Profile 2: Higher income (>10,000 yuan) was associated with lower odds of being in the High-barrier group (OR = 0.535, 95% CI: 0.305–0.938, *p* = 0.029). Compared with no pre-pregnancy exercise, daily exercise (OR = 0.332, 95% CI: 0.141–0.782, *p* = 0.012), and one to two times/week (OR = 0.437, 95% CI: 0.228–0.840, *p* = 0.013) were associated with lower odds of high barrier membership, whereas exercise three to six times per week was not (OR = 0.489, 95% CI: 0.221–1.080, *p* = 0.077). This pattern suggests a non-linear or threshold effect rather than a simple dose–response relationship. Receiving exercise guidance (OR = 0.364, *p* = 0.016) and higher GAD-7 scores (OR = 1.088, *p* = 0.011) were also significant predictors. Social support showed a borderline association (OR = 0.959, *p* = 0.058). No significant association was observed between education level and profile membership in either comparison (Profile 3 vs. Profile 2: OR = 0.687, 95% CI: 0.366–1.289, *p* = 0.242).

## 4 Discussion

### 4.1 Level of barriers to PA during pregnancy

The mean BPAPS item score was 2.51 ± 0.60, which is lower than the score of 3.05 reported in an Iranian sample, where the highest scores were for interpersonal barriers (Dolatabadi et al., 2022), but consistent with a previous Chinese study (Shang et al., 2023). The intrapersonal barriers related to the pregnancy subscale scored the highest (2.87 ± 0.73), followed by intrapersonal barriers unrelated to pregnancy (2.47 ± 0.78), environmental barriers (2.34 ± 0.67), and interpersonal barriers (2.14 ± 0.70). This pattern suggests that pregnancy-specific physiological changes, such as fatigue, gastrointestinal discomfort, and concerns about complications, are the most prominent barriers perceived by Chinese pregnant women, consistent with previous studies (Harrison et al., 2018; Sytsma et al., 2018). Therefore, individualized and targeted intervention measures should be formulated and implemented based on the different patterns of physical change.

### 4.2 Profiles of barriers to PA during pregnancy

This study identified three profiles of barriers to PA during pregnancy through LPA, namely “Low-barrier group” (10.3%), “Moderate-barrier group” (54.3%), and “High-barrier group” (35.3%). It also demonstrated that pregnant women exhibit group heterogeneity in their levels of barriers to PA during pregnancy.

The “Low-barrier group” comprised women who reported minimal barriers across all dimensions. Notably, even in this group, item 9 (“I am concerned with possible pregnancy complications such as miscarriages and premature labor”) had a relatively higher score, suggesting that safety concerns are nearly universal among pregnant women, regardless of overall barrier levels. This finding aligns with the qualitative literature indicating that fear of pregnancy complications is a pervasive concern (McKeough et al., 2022). The “Moderate-barrier group” represented the largest proportion of our sample. Women in this group reported moderate levels of barriers, with the highest scores concentrated in items related to pregnancy-related physical discomfort. The “High-barrier group” comprised more than one-third of our sample. This group was characterized by lower education and income, lack of pre-pregnancy exercise, absence of exercise guidance, low social support, and higher anxiety. However, the characteristics of the High-barrier group, such as income and educational level, have not been consistently reported (Dolatabadi et al., 2022; Shang et al., 2023). This inconsistency may be due to differences in study populations across regions.

### 4.3 Factors distinguishing the High-barrier group from the Moderate-barrier group

Higher income (>10,000 yuan) was significantly associated with lower odds of high barrier membership. This finding is consistent with the socioeconomic gradient in health behaviors, where higher income is associated with greater access to resources that facilitate PA, such as gym memberships, exercise equipment, and leisure time (Pampel et al., 2010). Several studies indicated that low socioeconomic status individuals may choose walking as a cost-saving alternative to motorized transportation (Todorovic et al., 2020); however, such a pattern is seldom observed within the Chinese context. However, the literature on income and PA barriers during pregnancy remains inconclusive. A study has reported that medium-income women had the highest barrier scores (Dolatabadi et al., 2022). This inconsistency may reflect differences in economic contexts and healthcare systems across countries.

Pre-pregnancy exercise frequency emerged as a robust predictor. Having regular exercise habits before pregnancy was associated with lower barriers to PA during pregnancy, compared with those with no pre-pregnancy exercise and those who exercised one to two times per week. This finding aligns with previous studies demonstrating that pre-pregnancy PA habits are protective against PA decline during pregnancy (Dolatabadi et al., 2022; Suberu and Adeoye, 2024). Habit formation theory suggests that once exercise behaviors become habitual, the underlying tendency facilitates behavior maintenance across life transitions (Verplanken et al., 1997). Habit-based interventions might be effective strategies for reducing PA barriers (Ma et al., 2023; McClelland et al., 2024). Notably, the comparison between Profile 1 and Profile 2 yielded wide confidence intervals, indicating imprecision in the estimates. This is likely attributable to the small cell counts in the cross-tabulation of pre-pregnancy exercise frequency and profile membership. Specifically, within the Low-barrier group (Profile 1, *n* = 31), only one participant reported no pre-pregnancy exercise. Despite this uncertainty, the direction and statistical significance of the effect were corroborated by the multinomial probit sensitivity analysis (d*y*/d*x* = −0.697, *p* = 0.014), supporting the robustness of the finding. Future studies with larger and more diverse samples are warranted to obtain more precise estimates for specific subgroups.

Receiving exercise guidance was also significantly associated with lower odds of being in the High-barrier group. This finding underscores the importance of healthcare provider counselling in mitigating PA barriers during pregnancy. Interestingly, the proportion of women who received guidance was higher in the Moderate-barrier group (17.8%) than in the Low-barrier group (9.7%), suggesting that receiving guidance alone may not suffice to eliminate barriers, which is consistent with studies showing that PA counselling from healthcare providers is often perceived as insufficient or ineffective (Brændstrup et al., 2023; Grenier et al., 2021). Some studies have revealed that PA counselling from healthcare providers is often perceived as minimal, generic, or even contradictory (Grenier et al., 2021; Lindqvist et al., 2014). This highlights the need for not only providing guidance but also ensuring its quality, individualized content, and continuity throughout pregnancy (Lindqvist et al., 2014; Santos-Rocha et al., 2022; Sparks et al., 2025).

Higher anxiety scores (GAD-7) were significantly associated with higher odds of high-barrier membership (OR = 1.088). This clinically relevant finding underscores the interplay between psychological distress and perceived barriers. Previous studies have revealed that low levels of anxiety were associated with the practice of exercise during pregnancy (Miranda et al., 2022), especially associated with moderate-to-vigorous intensity PA (Freitas et al., 2022). Pregnant women with higher anxiety may be more cautious about PA due to fears of harming the fetus or themselves, or they may experience reduced motivation and energy associated with anxiety symptoms. Our results extend this evidence by demonstrating that anxiety specifically differentiates women with high versus moderate barriers to PA. Integrated care models that address both mental health and physical activity may be particularly beneficial for pregnant women with elevated anxiety.

Social support showed a borderline association (OR = 0.959, *p* = 0.058). Although not statistically significant at the conventional *p* < 0.05 threshold, the trend suggests that higher social support may reduce the likelihood of being in the High-barrier group. Social support for pregnant women is important for maternal and infant health and wellbeing (Collins et al., 1993). This is consistent with the broader literature documenting social support as an enabler of PA during pregnancy (Chang et al., 2015; Da Costa and Ireland, 2013; McKeough et al., 2022). The lack of statistical significance may be attributable to supporters’ own knowledge and beliefs about PA, as well as the small sample size in the Low-barrier group (*n* = 31). Future studies with larger samples are needed to clarify the role of social support in distinguishing barrier profiles and to assess supports’ PA-related attitudes and behaviors.

## 5 Limitations

Several limitations should be acknowledged. First, the cross-sectional design precludes causal inferences; therefore, we can only identify associations rather than determine causality. Additionally, the predominance of third-trimester participants may have introduced selection bias. Longitudinal studies are needed to examine the changes across different gestational periods. Second, the sample was recruited from a single-center hospital in Shanghai, China, which may limit the generalizability of our findings to other regions or populations. Third, multiple univariate comparisons may have inflated the type I error rate. We did not apply Bonferroni or similar corrections to these descriptive analyses, as they were exploratory and intended to summarize sample characteristics rather than test *a priori* hypotheses (Rothman, 1990). However, the main regression findings were cross-validated using multinomial probit regression with robust standard errors, which yielded consistent results, supporting the robustness of our conclusions. Fourth, the small sample size in Profile 1 (*n* = 31) limited the statistical power to detect factors distinguishing this group from others. Fifth, to balance respondent burden and data quality, we assessed only one psychological factor (anxiety); other factors such as depression, self-efficacy, and motivation warrant future investigation. Sixth, the convenience sampling method may have introduced selection bias, and the study relied entirely on self-report measures, which are subject to recall bias and social desirability bias.

## 6 Conclusion

Barriers to PA among pregnant women are heterogeneous. This study identified three distinct latent profiles of barriers: a Low-barrier group (10.3%), a Moderate-barrier group (54.3%), and a High-barrier group (35.3%). Income, frequency of pre-pregnancy exercise, exercise guidance, and anxiety were significant factors associated with latent profile membership. Healthcare providers should assess these factors to quickly identify pregnant women who may have substantial barriers to PA. Future multicenter studies with larger samples and longitudinal designs are needed to confirm these findings and to evaluate the effectiveness of targeted interventions based on latent profile membership.

### 6.1 Relevance for clinical practice

Our findings have several practical implications. First, the identification of three distinct profiles enables healthcare providers to quickly screen pregnant women using simple questions, such as pre-pregnancy exercise frequency and income, to identify those at high risk of experiencing elevated PA barriers. Second, targeted interventions should be tailored to each profile: for the Moderate-barrier group, interventions should focus on managing pregnancy-related physical discomforts; for the High-barrier group, comprehensive approaches addressing multiple psychosocial and behavioral factors are needed. Third, healthcare systems should strengthen the quality of PA counselling, moving beyond generic advice to provide individualized, evidence-based guidance that addresses the specific concerns and barriers of each woman.

## Data Availability

The raw data supporting the conclusions of this article will be made available by the authors, without undue reservation.

## Footnotes

1
www.wjx.cn
