# Supplementary material for: Three distinct profiles of barriers to physical activity during pregnancy among Chinese women: a latent profile analysis
Source: Front Psychol. 2026 Jul 23;17:1883818. doi: 10.3389/fpsyg.2026.1883818 (PMC13441748; doi:10.3389/fpsyg.2026.1883818)
Supplement: Supplementary file 1 [file Data_Sheet_1.pdf]

Appendix Table A1. Robustness check: Average marginal effects from multinomial probit regression (baseline: Profile 2)

**Predictors**	**Profile 1 vs Profile 2**			**Profile 3 vs Profile 2**		
	dy/dx	Robust SE	p-value	dy/dx	Robust SE	p-value
**Education (ref: high school or below)**						
**College or more**	0.314	0.290	0.279	-0.283	0.256	0.269
**Income (ref: ≤10,000 yuan)**						
**>10,000 yuan**	-0.269	0.307	0.381	-0.541	0.236	**0.022**
**Pre-pregnancy exercise frequency (ref: no exercise)**						
**1-2 times/week**	0.910	0.542	0.093	-0.697	0.282	**0.014**
**3-6 times/week**	1.196	0.568	**0.035**	-0.586	0.337	0.082
**Daily**	1.097	0.580	0.059	-0.915	0.354	**0.010**
**Received exercise guidance (ref: no guidance)**						
**Yes**	-0.562	0.443	0.205	-0.877	0.337	**0.009**
**Social support rating scale score**	0.036	0.021	0.090	-0.032	0.018	0.069
**GAD-7 score**	0.009	0.041	0.829	0.068	0.027	**0.013**
**Constant**						
**Observations**		300			300	
**Log-likelihood**		-249.206				
**LR χ^2 (df)**		60.59(16)				
**P**		<0.001				

Note: OR = odds ratio; CI = confidence interval; dy/dx = average marginal effect; SE = standard error; GAD-7 = Generalized Anxiety Disorder Scale. Reference categories: Profile 2 for the dependent variable; "No exercise" for pre-pregnancy exercise frequency; "No guidance" for exercise guidance. Bold values indicate statistical significance at $p < 0.05$. All models estimated with robust standard errors (Huber-White). The two models yield consistent results for all key predictors, confirming robustness against IIA assumption violations.
